# Building an Evidence Base for the Co-Occurrence of Chronic Disease and Psychiatric Distress and Impairment

**DOI:** 10.5888/pcd11.140211

**Published:** 2014-10-23

**Authors:** Gina M. Piane, Tyler C. Smith

**Affiliations:** Author Affiliation: Tyler C. Smith, National University, San Diego, California.

## Abstract

**Introduction:**

Mental disorders and chronic diseases have been reported to independently affect half of the US population. The objective of this study was to evaluate the comorbid nature of these conditions.

**Methods:**

We analyzed data from 39,954 participants from the 2009 California Health Interview Survey who reported both psychological distress and impairment, on the basis of the Kessler 6 and the Sheehan Disability Scale, and 1 or more of 4 chronic diseases (type 2 diabetes, high blood pressure, asthma, heart disease). Weighted and nonweighted multivariable logistic regression were used to investigate the association between psychological distress and impairment and chronic disease, after adjusting for sex, age, race, current smoking, binge drinking in the previous year, moderate physical activity, and body mass index.

**Results:**

After controlling for covariates in the model, we found a significant dose–response relationship between reported chronic diseases and psychiatric distress and impairment that ranged from 1.50 for 1 reported chronic disease to 4.68 for 4 reported chronic diseases.

**Conclusion:**

The growing chronic disease burden should be understood clinically in the context of mental health conditions. Further research is needed to identify ways to integrate mental health and chronic disease prevention in primary care.

## MEDSCAPE CME

Medscape, LLC is pleased to provide online continuing medical education (CME) for this journal article, allowing clinicians the opportunity to earn CME credit.

This activity has been planned and implemented in accordance with the Essential Areas and policies of the Accreditation Council for Continuing Medical Education through the joint sponsorship of Medscape, LLC and Preventing Chronic Disease. Medscape, LLC is accredited by the ACCME to provide continuing medical education for physicians.

Medscape, LLC designates this Journal-based CME activity for a maximum of 1 **AMA PRA Category 1 Credit(s)™**. Physicians should claim only the credit commensurate with the extent of their participation in the activity.

All other clinicians completing this activity will be issued a certificate of participation. To participate in this journal CME activity: (1) review the learning objectives and author disclosures; (2) study the education content; (3) take the post-test with a 75% minimum passing score and complete the evaluation at www.medscape.org/journal/pcd (4) view/print certificate.

**Release date: October 23, 2014; Expiration date: October 23, 2015**

### Learning Objectives

Upon completion of this activity, participants will be able to:

- Analyze the epidemiology of chronic medical disease and mental illness among adults in the United States.
- Evaluate the association between chronic disease and psychiatric distress in the current study.
- Assess demographic variables that may strengthen the association between chronic disease and psychiatric distress.
- Assess other variables that may strengthen the association between chronic disease and psychiatric distress.

**EDITORS**

Rosemarie Perrin, Editor, *Preventing Chronic Disease*. Disclosure: Rosemarie Perrin has disclosed no relevant financial relationships.

**CME AUTHOR**

Charles P. Vega, MD, Clinical Professor of Family Medicine, University of California, Irvine. Disclosure: Charles P. Vega, MD, has disclosed the following relevant financial relationships: Served as an advisor or consultant for: McNeil Pharmaceuticals.

**AUTHORS AND CREDENTIALS**

Disclosures: Gina M. Piane, DrPH, MPH, and Tyler C. Smith have disclosed no relevant financial relationships.

Affiliations: Gina M. Piane, DrPH, MPH, Department of Community Health, School of Health and Human Services, National University, San Diego, California; Tyler C. Smith, PhD, MS, National University, San Diego, California.

## Introduction

In the past century, chronic diseases have surpassed infectious diseases as the leading cause of death in the United States. Nearly half of the US population lives with at least 1 chronic disease, and 7 out of 10 deaths have been attributed to these diseases (1–3). Cardiovascular disease is a major contributor to these deaths; however, escalating obesity prevalence has led to an epidemic of type 2 diabetes. Projections are that 5.4% percent of the world’s adult population will have been diagnosed with this disease by 2025, including an estimated 48 million Americans (4). This trend concerns public health professionals and health care providers as they prepare for treatment and control of this increasing chronic disease burden. Of perhaps greater concern is the growing number of people with multiple chronic conditions, including mental health disorders. Preventing, diagnosing, and treating disease and integrating health care delivery to address comorbidities will help to define health care successes in this century.

Diagnosis and treatment of mental health disorders have been the focus of research in the past 2 decades; medical professionals are attempting to understand the nature of these disorders and how they affect other health conditions and overall health (5–9). Reports published in 2005 suggested that more than 1 in 4 Americans aged 18 years or older will experience a diagnosable mental disorder in any given year and that nearly half will experience a mental health disorder in their lifetimes (9,10). The estimated cost in 2002 associated with this burden of mental illness was $300 billion (11). Much of the desire for increased understanding of mental health disorders resulted from the wars in Iraq and Afghanistan in the past 10 years and the mental health symptoms associated with US combat deployment. Reports from military populations suggested that decrements in sleep and functional health are associated with chronic diseases such as diabetes and hypertension (12–14).

The Institute of Medicine’s (IOM) reports, *Crossing the Quality Chasm: A New Health System for the 21st Century* (15) and *Improving the Quality of Health Care for Mental and Substance-Use Conditions* (16) urged the US health care system to integrate the provision of mental health and primary care services. The IOM identified the lack of mental health and substance use care as a pervasive problem in the US health care system and recommended that health services be delivered with an understanding of the interaction of mental and general health needs.

The US Veterans Administration (VA) and several university health services successfully implemented health care delivery guided by the IOM’s vision of integrated mental health and primary care (17–20). The VA’s Primary Care–Mental Health Integration program resulted in elevated diagnosis patterns of mental disorders (19). Similarly, several university health care systems demonstrated the feasibility of integrated mental health and primary care by documenting both clinical improvements and financial benefits from using an integrated approach (20). These results, however, cannot be generalized to community care settings without further study.

The Centers for Disease Control and Prevention’s (CDC’s) National Center for Chronic Disease Prevention and Health Promotion (NCCDPHP) developed a public heath action plan to integrate mental health promotion, mental illness prevention, and chronic disease prevention (21). The plan recommends increased surveillance of mental health and chronic disease measures, epidemiology research, prevention research, and more communication among health professionals and the public. These efforts support the mission of the NCCDPHP, which is to prevent death and disability from chronic disease and to promote healthy behaviors. Mental disorders are among the most prevalent and costly conditions in the United States, and effective treatment can reduce their prevalence and decrease their adverse effect on chronic conditions. Integrating mental health and public health programs that address chronic disease is essential to protecting the health of Americans (21).

The evolving disease burden in the United States along with a growing understanding of disease comorbidities and risk factors necessitates a continuum of care that integrates all aspects of health care. Because psychiatric distress and impairment are likely influenced by chronic disease diagnosis and maintenance, it is important to understand the relationship between these health concerns, which are often clinically disconnected. The objective of our study was to estimate the association between psychiatric distress and impairment and chronic diseases while controlling for known risk factors by using data from a large cross-sectional study of Californians.

## Methods

### Population and data sources

Participants included in this study participated in the 2009 California Health Interview Survey (CHIS), maintained by the University of California, Los Angeles (UCLA) Center for Health Policy Research under IRB protocol 09–05-103–02, a population-based telephone survey of Californians that has been conducted every 2 years since 2001. A multistage sampling design was used to sample both land line and cellular telephone service subscribers throughout the state. Within each household, 1 adult aged 18 years or older was randomly selected to participate. Interviews were conducted in 5 languages: English, Spanish, Chinese (Mandarin and Cantonese dialects), Vietnamese, and Korean; these languages were chosen on the basis of analysis of 2000 census data indicating that these languages would cover the largest number of Californians. Advance letters and incentives were used to enhance participation. Although the overall response rate for the adult data set was approximately 1 in 4, nonresponse bias was not shown to affect the representativeness of the data. This research was conducted in compliance with all applicable federal regulations governing the protection of human subjects in research. The National University institutional review board reviewed this research and granted it exempt status because it analyzed existing data.

### Chronic disease assessment

Chronic disease was assessed by using participants’ affirmative response to a question asking whether a doctor ever told them they had 1 or more of the following conditions: type 2 diabetes, high blood pressure, asthma, and any kind of heart disease. Respondents who indicated type 1 diabetes or other diabetes were excluded from these analyses. Affirmative responses for these 4 conditions were combined to form an aggregate variable. A count variable was also calculated that summed the number of chronic diseases reported so that effect of chronic disease comorbidity could be assessed.

### Psychiatric distress and impairment

The psychiatric distress and impairment variable was calculated as an aggregate variable to include serious psychological distress and impairment. The Kessler 6 (K6) is a self-report, 6-item scale measuring feelings of nervousness, hopelessness, restlessness, depression, everything being an effort, and worthlessness and was used to assess nonspecific psychological distress (22). The K6 scale is internally consistent and reliable (Cronbach’s α = 0.89), has consistent psychometric properties across major sociodemographic subsamples, and discriminates well between community cases and noncases of Diagnostic and Statistical Manual/Structured Clinical Interview for DSM Disorders (DSM-IV/ SCID) disorders as determined by the areas under the receiver operating characteristic curve (22). Participants with a sum of 13 or higher of 24 were categorized as having serious psychological distress.

Moderate to severe impairment was assessed through any moderate or severe report of components included in the Sheehan Disability Scale (23). This 4-item scale captures impairment in 4 life domains including chores, family life, and social life (23,24). Moderate to severe impairment was combined with psychological distress and evaluated as an aggregate variable.

### Covariates

Additional covariates were assessed through self-report. Age was categorized as 18 to 24, 25 to 39, 40 to 64, and 65 or older. Current smoking was assessed with a participant’s affirmative response when asked whether they currently smoked. Binge drinking was categorized as drinking 5 or more drinks in a 24-hour period for men and 4 or more drinks in a 24-hour period for women anytime in the past year. Moderate physical activity in the past 7 days was assessed with an affirmative response when participants were asked if they had been moderately active in the past week (“During the last 7 days, did you do any moderate physical activities in your free time for at least 10 minutes, other than walking?”). Race/ethnicity was categorized as non-Hispanic white, non-Hispanic black, Hispanic, Asian, and other. Body mass index (BMI [kg/m^2^]) was categorized as underweight (<18.5), normal (18.5–24.9), overweight (25.0–29.9), or obese (≥30.0).

### Statistical analyses

Descriptive and univariate analyses were used to evaluate the unadjusted associations between the aggregated chronic disease and the chronic disease count variables, the psychiatric distress and impairment aggregate variable, and other covariates. The CHIS employs a 2-stage geographically stratified random digit-dial sample design, which necessitates weighting of the data (25). Provided weights account for sample selection probabilities, nonresponse biases, and other adjustments designed for valid variance estimation. Weighted χ^2^ test statistics were used to calculate *P* values and are reported. A multivariable exploratory model analysis was conducted to assess multicollinearity, significant associations, and possible confounding while adjusting for all other covariates in the model. Multivariable logistic regression was used to compare the differences in adjusted odds of psychiatric distress and impairment while controlling for possible confounders. Additionally, weighted logistic regression models were built where the intersection of psychiatric distress and impairment and chronic disease or any chronic disease was the end point. We used SAS version 9.2 (SAS Institute, Inc) to calculate weighted and nonweighted (based on sampling and response) odds ratios (ORs) and 95 percent confidence intervals (CIs) for participants with complete covariate data. Significance was set at *P* <.10.

## Results

Of the 47,614 adult participants in the CHIS 2009 survey, 39,954 (84%) had complete data for the variables investigated in this analysis. Of the 39,954 participants investigated, 59% were female, 47% were aged 40 to 64 years, 68% were white, and 89% were nonsmokers. Seventy-eight percent said they were not binge drinkers, 58% reported moderate exercise, and 56% were overweight or obese (Table 1).

**Table 1:** Demographic Characteristics of California Health Interview Survey (CHIS) Participants Aged 18 Years or Older Who Reported Having Type 2 Diabetes, High Blood Pressure, Asthma, or Heart Disease, 2009

Characteristic	CHIS participants (N = 39,954), n (%)^a^	Participants Reporting at Least 1 Chronic Disease (N = 19,449), n (%)^a^	*P* Value^b^
**Sex**			
Male	16,491 (41.3)	8,219 (42.3)	.23
Female	23,463 (58.7)	11,230 (57.7)	
**Age, y**			
18–24	1,918 (4.8)	458 (2.4)	<.001
25–39	5,161 (12.9)	1,184 (6.1)	
40–64	18,899 (47.3)	8,078 (41.5)	
≥65	13,976 (35.0)	9,729 (50.0)	
**Race/ethnicity**			
Non-Hispanic white	27,083 (67.8)	13,923 (71.6)	<.001
Non-Hispanic black	1,622 (4.1)	1,005 (5.2)	
Hispanic	4,603 (11.5)	1,712 (8.8)	
Asian	4,063 (10.2)	1,595 (8.2)	
Other	2,583 (6.5)	1,214 (6.2)	
**Current smoker**			
Yes	4,216 (10.6)	1,972 (10.1)	.98
No	35,738 (89.5)	17,477 (89.9)	
**Binge drinking in previous year^c^**			
Yes	8,883 (22.2)	3,579 (18.4)	<.001
No	31,071 (77.8)	15,870 (81.6)	
**Moderate physical activity^d^ in the past 7 days**			
Yes	23,298 (58.3)	10,830 (55.7)	.13
No	16,656 (41.7)	8,619 (44.3)	
**Body mass index (kg/m^2^)**			
Underweight (<18.5)	872 (2.2)	326 (1.7)	<.001
Normal (18.5–24.9)	16,692 (41.8)	6,454 (33.2)	
Overweight (25.0–29.9)	13,712 (34.3)	7,074 (36.4)	
Obese (≥30.0)	8,678 (21.7)	5,595 (28.8)	

a Percentages may not sum to total because of rounding.

b *P* values are based on Pearson χ^2^ test of association using sampling weights for variance estimation.

c Defined as 5 or more drinks on 1 occasion for men and 4 or more drinks for women.

d Defined as engaging in any moderate physical activity other than walking for at least 10 minutes in the last 7 days.

We assessed demographic characteristics by whether participants reported at least 1 of the 4 chronic diseases investigated in this analysis (Table 1). Age, race/ethnicity, binge drinking, and BMI were significantly associated with reported chronic disease. Older, white, overweight participants were proportionately more likely to report at least 1 of the 4 chronic diseases.

Reported chronic diseases, sex, age, race/ethnicity, current smoking, binge drinking, and moderate physical activity were significantly associated with any psychiatric distress and impairment (Table 2). Participants reporting more chronic diseases, women, younger participants, Hispanics, current smokers, binge drinkers, those not reporting moderate physical activity, and obese participants were proportionately more likely to report psychiatric distress and impairment.

**Table 2:** Demographic Characteristics of Participants Aged 18 Years or Older Reporting Chronic Disease^a^ With or Without Psychiatric Distress and Impairment, California Health Interview Survey, 2009

Characteristic	Participants Reporting No Psychiatric Distress and Impairment (N = 36,643), n (%)^b^	Participants Reporting Psychiatric Distress and Impairment (N = 3,311), n (%)^b^	*P* Value^c^
**No. chronic diseases^a^ reported^c^**			
0	18,920 (51.6)	1,585 (47.9)	.02
1	12,034 (32.8)	1,082 (32.7)	
2	4,495 (12.3)	472 (14.3)	
3	1,087 (3.0)	142 (4.3)	
4	107 (0.3)	30 (0.9)	
**Sex**			
Male	15,322 (41.8)	1,169 (35.3)	.047
Female	21,321 (51.2)	2,142 (64.7)	
**Age, y**			
18–24	1,691 (4.6)	227 (6.9)	<.001
25–39	4,607 (12.6)	554 (16.7)	
40–64	16,946 (46.3)	1,953 (59.0)	
≥65	13,399 (36.6)	577 (17.4)	
**Race/ethnicity**			
Non-Hispanic white	24,918 (68.0)	2,165 (65.4)	.08
Non-Hispanic black	1,473 (4.0)	149 (4.5)	
Hispanic	4,186 (11.4)	417 (12.6)	
Asian	3,753 (10.2)	310 (9.4)	
Other	2,313 (6.3)	270 (8.2)	
**Current smoker**			
Yes	3,356 (9.7)	680 (20.5)	<.001
No	33,107 (90.4)	2,631 (79.5).)	
**Binge drinking^d^ in previous year**			
Yes	7,919 (21.6)	964 (29.1)	.02
No	28,724 (78.4)	2,347 (70.9)	
**Moderate physical activity^e^ in the past 7 days**			
Yes	21,588 (58.9)	1,710 (51.7)	.09
No	15,055 (41.1)	1,601 (48.4)	
**Body mass index (kg/m^2^)**			
Underweight (<18.5)	787 (2.2)	85 (2.6)	.42
Normal (18.5–24.9)	15,359 (41.9)	1,333 (40.3)	
Overweight (25.0–29.9)	12,699 (34.7)	1,013 (30.6)	
Obese (≥30.0)	7,798 (21.3)	880 (26.7)	

a Chronic diseases assessed were type 2 diabetes, high blood pressure, asthma, and heart disease.

b Percentages may not sum to total because of rounding.

c *P* values are based on Pearson χ^2^ test of association using sampling weights for variance estimation.

d Defined as 5 or more drinks on 1 occasion for men and 4 or more drinks for women.

e Defined as engaging in any moderate physical activity other than walking for at least 10 minutes in the last 7 days.

We assessed multicollinearity and found no variables with a variance inflation level at or greater than 4. We also assessed weighted and nonweighted adjusted multivariable logistic regression analysis results (Table 3). Because weighted and nonweighted measures were consistent, we elected to consider only weighted measures. After controlling for sex, age, race/ethnicity, current smoking, binge drinking, moderate physical activity, and BMI we found a dose–response relationship between reported chronic diseases and psychiatric distress and impairment. Odds ratios for the measures of effect ranged from 1.50 for 1 reported chronic disease (1.5 times the odds of psychiatric distress and impairment for 1 reported chronic disease) to 4.68 for 4 reported chronic diseases. The 95% CIs for these adjusted and weighted odds ratios were all significant. Independent of the covariates included in the model, women were 1.39 times more likely to report psychiatric distress and impairment (95% CI = 1.15–1.69) than men. Smokers were 1.95 times more likely to report psychiatric distress and impairment (95% CI = 1.51–2.52) than nonsmokers, and participants reporting moderate physical activity were 0.79 less likely to report psychiatric distress and impairment (95% CI = 0.65–0.95) than those not reporting moderate physical activity.

**Table 3:** Adjusted Odds of Reporting Psychiatric Distress and Impairment and a Chronic Disease^a^ Calculated by Logistic Regression in Participants Aged 18 Years or Older, California Health Interview Survey, 2009

Characteristic	Nonweighted Adjusted Odds of Reporting Psychiatric Distress and Impairment, AOR (95% CI)	Weighted Adjusted Odds of Reporting Psychiatric Distress and Impairment, AOR (95% CI)
**No. chronic diseases^a^ reported^b^**		
0	1 [Reference]	
1	1.35 (1.24–1.47)	1.50 (1.24–1.83)
2	1.96 (1.74–2.21)	1.74 (1.29–2.36)
3	2.60 (2.14–3.17)	2.51 (1.37–4.60)
4	5.66 (3.68–8.70)	4.68 (2.45–8.93)
**Sex**		
Male	1 [Reference]	
Female	1.45 (1.35–1.57)	1.39 (1.15–1.69)
**Age, y**		
18–24	1 [Reference]	
25–39	0.84 (0.71–0.99)	1.09 (0.81–1.48)
40–64	0.73 (0.63–0.85)	0.84 (0.63–1.12)
≥65	0.24 (0.20–0.29)	0.35 (0.24–0.51)
**Race/ethnicity**		
Non-Hispanic white	1 [Reference]	
Non-Hispanic black	0.92 (0.77–1.10)	1.21 (0.80–1.83)
Hispanic	0.89 (0.79–1.00)	0.83 (0.66–1.05)
Asian	0.88 (0.77–1.00)	0.84 (0.57–1.22)
Other	1.04 (0.91–1.20)	1.48 (1.01–2.17)
**Current smoking**		
Yes	2.13 (1.93–2.34)	1.95 (1.51–2.52)
No	1 [Reference]	
**Binge drinking^c^ in previous year**		
Yes	1.23 (1.12–1.32)	1.20 (0.96–1.51)
No	1 [Reference]	
**Moderate physical activity^d^ in the past 7 days**		
Yes	0.79 (0.74–0.85)	0.79 (0.65–0.95)
No	1 [Reference].	
**Body mass index (kg/m^2^)**		
Underweight (<18.5)	1.16 (0.91–1.46)	1.09 (0.57–2.09)
Normal (18.5–24.9)	1 [Reference]	
Overweight (25.0–29.9)	0.92 (0.84–1.00)	0.92 (0.75–1.13)
Obese (≥30.0)	1.08 (0.98–1.19)	1.00 (0.80–1.24)

Abbreviations: AOR, adjusted odds ratio; CI, confidence interval.

a Chronic diseases assessed were type 2 diabetes, high blood pressure, asthma, and heart disease.

b Percentages may vary because of rounding.

c Defined as 5 or more drinks on 1 occasion for men and 4 or more drinks for women.

d Defined as engaging in any moderate physical activity other than walking for at least 10 minutes in the last 7 days.

## Discussion

Chronic disease burden continues to grow in the United States. At any given time, an estimated half of the population has 1 of the 4 chronic diseases we studied (1–3). Diagnosable mental health disorders affect 1 in 4 US adults, and it is estimated that one-half of the population will experience at least 1 mental health disorder in their lifetimes leading to substantial cost to the health care system (9–11). These numbers indicate a significant, though often misunderstood, intersection of mental and physical disorders. We found a significant association between chronic disease and psychiatric distress and impairment independent of other known risk factors (ie, sex, age, race, current smoking, binge drinking, moderate physical activity, and BMI). Participants with increasing numbers of reported chronic disease presented a graded increase in adjusted odds of psychiatric distress or impairment. Adjusting for chronic disease reporting in this study, being younger, female, a smoker, a binge drinking, or engaging in less physical activity were associated with increased adjusted odds of psychiatric distress and impairment. However, BMI was not found to be associated with psychiatric distress and impairment independent of chronic disease, which is consistent with Fabricatore and Wadden’s research (26) and may explain other studies that have found this association in the absence of chronic disease assessment (27). Race/ethnicity was not significantly associated with an increase in adjusted odds of psychiatric distress and impairment, and studies present a varied picture of the potential association. Some studies found a difference in the association between race/ethnicity and psychiatric distress and impairment (28), and others are less definitive and suggest alternative reasons for apparent differences (29). An understanding of psychiatric distress and impairment across race/ethnicity groups in the context of chronic disease may help to isolate these findings and create more consistency in reporting.

Consistent with findings from other studies, we found that older, white, and obese respondents were more likely to report at least 1 chronic disease (30). However, those with both psychiatric distress and impairment and chronic disease have different characteristics than the risk factors for chronic disease and psychiatric distress and impairment separately. In this intersection, Participants who were overweight or obese, non-Hispanic black, older, and who smoked had the heaviest burden of chronic disease and psychiatric distress and impairment. Failure to address the burgeoning needs of this hard-to-reach, highest-risk population will have a significant effect on the US health care system now and in the future. The choice we have is whether the aging of our population will increase the years of vibrant and productive life or will diseases of aging burden the health care system with the need to control and treat chronic diseases in ever growing numbers of older adults. Our findings identify subgroups of the US population that may benefit from focused screening in primary care.

Implementation of the NCCDPHP’s public heath action plan to integrate mental health promotion, mental illness prevention and chronic disease prevention (21) should address the needs of these high-risk Americans. A needs assessment must include older, obese, non-Hispanic blacks who smoke. The dose–response relationship between reported chronic diseases and psychiatric distress and impairment is also significant to the health care planning process. Treating chronic disease and mental health in silos will not address the burden of comorbidities in high-risk populations.

Our study has limitations. First, our data were cross-sectional, which did not allow temporal sequence to be investigated and yielded only associations without the ability to address risk. These data may not be generalizable to the entire US population and do not include information from institutionalized populations or people without telephones. Self-reported health outcomes are an imperfect surrogate for physician diagnosis and may result in misclassification of both psychiatric distress and impairment and chronic disease status.

Using data from this large cross-section of Californians had several strengths. First and foremost, these analyses were possible because of the public use data files made available to researchers by the UCLA Center for Health Policy Research. The size and scope of the CHIS and the inclusion of many racial/ethnic minorities helped to make our inferences generalizable. Several potential confounders and risk factors for mental health disorders were available and used for these analyses.

Psychiatric distress and impairment and chronic diseases frequently coexist, and people with a greater number of chronic conditions demonstrated greater need for mental health services. Consistent with the IOM’s recommendations to integrate mental health services with primary care, our findings suggest that integrating mental health with primary care could result in more consistent diagnosis of mental disorders and more opportunities for prevention and treatment. Epidemiologic studies like ours that advance knowledge of coexistence of psychiatric distress and impairment and chronic disease support CDC’s action plan for bridging mental health and public health, leading to improved treatment and reduced treatment cost for these disorders.

## Figures and Tables

**Table:** 

**1. The activity supported the learning objectives.**				
**Strongly Disagree**				**Strongly Agree**
1	2	3	4	5
**2. The material was organized clearly for learning to occur.**				
**Strongly Disagree**				**Strongly Agree**
1	2	3	4	5
**3. The content learned from this activity will impact my practice.**				
**Strongly Disagree**				**Strongly Agree**
1	2	3	4	5
**4. The activity was presented objectively and free of commercial bias.**				
**Strongly Disagree**				**Strongly Agree**
1	2	3	4	5

## Post-Test Information

To obtain credit, you should first read the journal article. After reading the article, you should be able to answer the following, related, multiple-choice questions. To complete the questions (with a minimum 75% passing score) and earn continuing medical education (CME) credit, please go to http://www.medscape.org/journal/pcd. Credit cannot be obtained for tests completed on paper, although you may use the worksheet below to keep a record of your answers. You must be a registered user on Medscape.org. If you are not registered on Medscape.org, please click on the "Register" link on the right hand side of the website to register. Only one answer is correct for each question. Once you successfully answer all post-test questions you will be able to view and/or print your certificate. For questions regarding the content of this activity, contact the accredited provider, CME@medscape.net. For technical assistance, contact CME@webmd.net. American Medical Association's Physician's Recognition Award (AMA PRA) credits are accepted in the US as evidence of participation in CME activities. For further information on this award, please refer to http://www.ama-assn.org/ama/pub/about-ama/awards/ama-physicians-recognition-award.page. The AMA has determined that physicians not licensed in the US who participate in this CME activity are eligible for **AMA PRA Category 1 Credits**™. Through agreements that the AMA has made with agencies in some countries, AMA PRA credit may be acceptable as evidence of participation in CME activities. If you are not licensed in the US, please complete the questions online, print the AMA PRA CME credit certificate and present it to your national medical association for review.

## Post-Test Questions

### Article Title: Building an Evidence Base for the Co-Occurrence of Chronic Disease and Psychiatric Distress and Impairment

**CME Questions**

1. You are seeing a 68-year-old African American woman with a history of hypertension, type 2 diabetes mellitus, and congestive heart failure. She reports feeling fatigue and poor motivation to perform even simple tasks. On further questioning, she meets the criteria for major depressive disorder. What should you consider regarding the epidemiology of chronic medical and mental health disorders as you evaluate this patient?
  - The prevalence of any chronic disease among US adults is approximately 20%
  - Most deaths are a result of chronic disease
  - The annual prevalence of mental health disorders among US adults is 2%
  - The lifetime prevalence of any mental health disorder among US adults is 15%
2. The patient is surprised to hear that she has symptoms consistent with depression. What was the relationship between chronic disease and psychiatric distress and impairment in the current study by Piane and Smith?
  - There was no significant relationship between the presence of chronic disease and psychiatric distress
  - There was a dose-response relationship between more chronic diseases and greater psychiatric distress
  - Only adults with at least 3 chronic diseases had significant elevations of psychiatric distress
  - Only adults with at least 4 chronic diseases had significant elevations of psychiatric distress
3. Which of the following demographic variables was ***most*** associated with a higher rate of psychiatric distress and impairment related to chronic disease in the current study?
  - Female gender
  - Nonwhite race
  - Older age
  - Lower socioeconomic status
4. Which of the following other variables was ***most*** associated with a higher rate of psychiatric distress and impairment related to chronic disease in the current study?
  - Cigarette smoking
  - Higher levels of physical activity
  - Higher body mass index
  - Race/ethnicity

**Evaluation**
